# Stereoselective Organic Reactions in Heterogeneous Semiconductor Photocatalysis

**DOI:** 10.3389/fchem.2019.00630

**Published:** 2019-09-18

**Authors:** Shigeru Kohtani, Akira Kawashima, Hideto Miyabe

**Affiliations:** School of Pharmacy, Hyogo University of Health Sciences, Kobe, Japan

**Keywords:** enantioselective reactions, diastereoselective reactions, semiconductor photocatalysis, heterogeneous photocatalysis, titanium dioxide, chiral reagents

## Abstract

The most significant feature of heterogeneous semiconductor photocatalysis is that both oxidation and reduction occur in a one-pot process. Thus, photocatalysis leads to unique redox organic reactions that cannot be achieved by conventional techniques using oxidants or reductants. Semiconductor photocatalysis is expected to be a new method for fine chemical syntheses of highly valuable molecules such as chiral medicines. However, the use of semiconductor photocatalysts in stereoselective reactions has been limited so far. This mini-review highlights recent progress in stereoselective organic reactions using semiconductor photocatalysts, briefly summarizing the enantio- and diastereoselective reactions based on the currently available literature.

## Introduction

Chirality is a fundamentally important topic in science because biologically active species such as enzymes selectively recognize a single enantiomer. Therefore, asymmetric synthesis has attracted extensive attention not only in organic chemistry but also in the medicinal, pharmaceutical and agricultural sciences. Up to now, highly stereoselective synthesis of chiral compounds has been made on a plant scale by conventional stereo-controlling methods (Crawley and Trost, 2012).

Heterogeneous photocatalysis on semiconductors is a unique redox methodology compared to traditional techniques using oxidants or reductants. Such photocatalysis, especially using titanium dioxide (TiO_2_), has attracted much attention in many fields (Fujishima et al., 2000). Despite significant advances, little attention has been focused on the use of semiconductors for synthetic organic chemistry (Fox, 1987; Fagnoni et al., 2007; Shiraishi and Hirai, 2008; Kohtani and Miyabe, 2014; Lang et al., 2014a,b; Kisch, 2017; Kou et al., 2017; Ma et al., 2018). Semiconductor photocatalysis has several great advantages (Kohtani et al., 2012): (1) It leads to unique one-pot redox transformations. (2) Particular reductants or oxidants are not necessary. (3) It avoids the use of dangerous and harmful reagents. (4) It proceeds under mild conditions (normal temperature and pressure). (5) Semiconductors such as TiO_2_ are chemically stable, easily separable, and reusable. Thus, such photocatalysis shows great promise to become “green” chemical processes. Moreover, semiconductor photocatalysis is expected to grow as a new synthetic method for preparing highly valuable molecules such as chiral medicines.

Enantioselective synthesis using homogeneous photocatalysts such as chiral metal complexes has attracted wide attention in recent years (Amador and Yoon, 2016; Megan et al., 2016). However, the use of semiconductors in asymmetric synthesis has been limited so far. To our knowledge, ~10 reports have been published on successful examples of enantio- and diastereoselective reactions using semiconductor photocatalysts. This mini-review highlights the progress in stereoselective chemical transformations on photoirradiated surfaces of semiconductor particles, briefly summarizing representative examples of enantio- and diastereoselective reactions based on the currently available literature.

## Enantioselective Reactions

**Figure 1:**
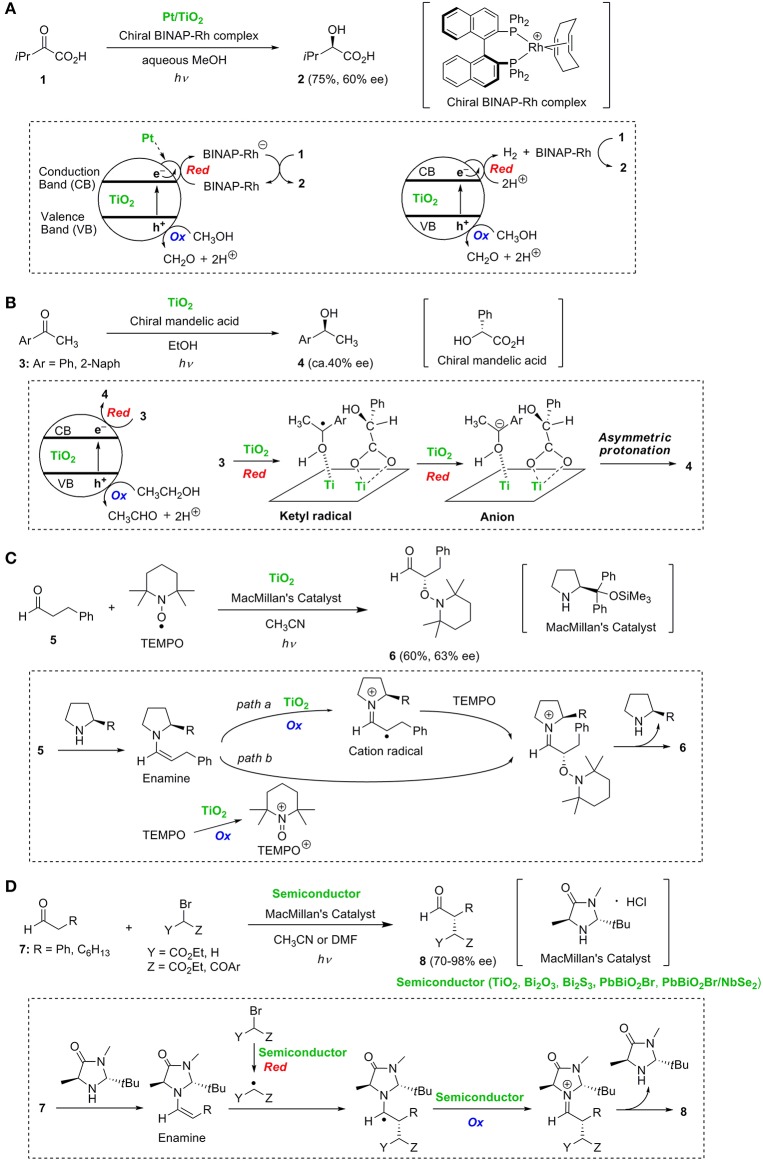
Enantioselective reactions: **(A)** hydrogenation of α-keto acid **1**, **(B)** hydrogenation of aromatic ketones **3**, **(C)** α-oxyamination of aldehyde **5**, and **(D)** α-alkylation of aldehydes **7**.

In 1990, Wang et al. (1990) reported that the enantioselective photoreduction of 3-methyl-2-oxobutanoic acid **1** proceeded in aqueous methanol suspension containing platinum loaded TiO_2_ (Pt/TiO_2_) and chiral 2,2'-bis(diphenylphosphino)-1,1'-binaphthyl (BINAP)-Rh complex to give 2-hydroxy-3-methylbutanoic acid **2** in 75% yield and 60% ee (Figure 1A). The mechanism involved in this asymmetric induction is still unclear. In this transformation, the photo-generated conduction band (CB) electrons must migrate toward Pt on TiO_2_. A route involving the transfer of electrons accumulated on Pt to the chiral BINAP-Rh complex and the subsequent reduction of **1** by the BINAP-Rh anion is proposed as a possible mechanism. Chiral metal catalysts possessing BINAP ligands are known to be efficient catalysts for the enantioselective hydrogenation of various olefinic and ketonic substrates in the presence of gaseous hydrogen (H_2_) in the dark (Shimizu et al., 2007). Accumulated electrons on Pt will reduce protons (H^+^) to produce H_2_. Therefore, it is possible that substrate **1** is reduced by gaseous H_2_ and the chiral BINAP-Rh complex as an alternate mechanism.

Kohtani et al. (2018) reported the novel surface-selective and enantioselective hydrogenation of aromatic ketones **3** induced by chiral α-hydroxy acids-coadsorbed on TiO_2_. When (*R*)-mandelic acid was used as the chiral reagent, the *S*-enantiomers of secondary alcohols **4** were predominantly obtained with reasonable enantioselectivities (ca. 40% ee) (Figure 1B). The enantioselectivities were strongly affected by the chiral reagents. Chiral mandelic acids having hydroxyl, phenyl and carboxy groups at the chiral carbon atom showed the best enantioselective stereocontrol. Interestingly, P25 (anatase/rutile = ca. 9/1) and an anatase TiO_2_ sample (JRC-TIO-13) exhibited relatively high enantioselectivities, whereas another anatase TiO_2_ (JRC-TIO-7) and the rutile TiO_2_ led to low % ee. Thus, the enantioselectivity was affected by the TiO_2_ crystalline samples. The reduction of ketones **3** on TiO_2_ proceeds *via* electron transfer to **3** leading to a ketyl radical species and further electron transfer to form an anion (Kohtani et al., 2014). Asymmetric induction is achieved through stereoselective protonation of the anion species by (*R*)-mandelic acid co-adsorbed on the TiO_2_ surface.

Jang's group reported TiO_2_-induced enantioselective α-oxyamination of aldehyde **5** with 2,2,6,6-tetramethylpiperidine-*N*-oxyl (TEMPO) by the use of a chiral amine catalyst (Figure 1C) (Ho et al., 2011). This reaction proceeds *via* a chiral enamine intermediate, generated from aldehyde **5** and MacMillan's catalyst, to give oxyamination product **6** in 60% yield with 63% ee. Two pathways are proposed for the oxidative transformation of the enamine to the iminium cation intermediate. The first pathway involves the oxidation of the enamine to a cation radical followed by stereoselective trapping of the cation radical by TEMPO (*path a* in Figure 1C). The second pathway involves the stereoselective reaction of the enamine with a cation species (TEMPO^+^) generated by the oxidation of TEMPO (*path b* in Figure 1C). The successful application to a tandem Michael addition-oxyamination was reported using N719 dye-sensitized TiO_2_ photocatalyst under visible light irradiation (Yoon et al., 2012).

Cherevatskaya et al. (2012) reported visible-light promoted enantioselective alkylation of aldehydes **7** by the use of several semiconductors and MacMillan's catalyst (Figure 1D). Later, Riente et al. (2014) achieved highly enantioselective α-alkylation of **7** using bismuth-based semiconductor materials (Bi_2_O_3_ and Bi_2_S_3_) possessing a small band gap and MacMillan's catalyst under sunlight (Figure 1D). Li et al. (2015) studied enantioselective alkylation of aldehydes using a nanocomposite material of PbBiO_2_Br nanoparticles with a NbSe_2_ nanosheet. The key step in these reactions is presumed to be the stereoselective addition of alkyl radicals to the chiral enamine intermediates.

Shi et al. (2014) developed an enantioselective molecular imprinting technique for photoelectrochemical and photocatalytic recognition of enantiomers. They reported the chiral recognition and enantioselective decomposition of amino acids on chiral molecular-imprinted ZnO and anatase TiO_2_ crystallites. Interestingly, the use of anatase TiO_2_ crystallites with specifically exposed (001) facets led to higher enantioselective recognition, presumably caused by abundant surface hydroxyls on the (001) facet (Shi et al., 2014).

## Diastereoselective Reactions and Asymmetric Synthesis

**Figure 2:**
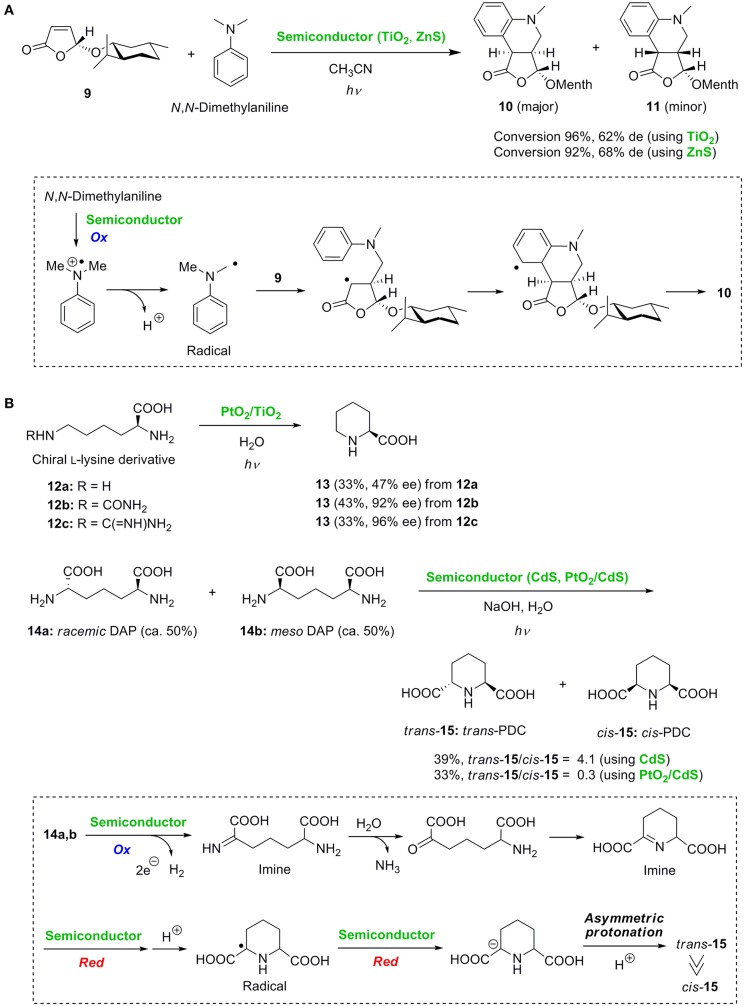
**(A)** Diastereoselective tandem radical addition-cyclization reaction and **(B)** asymmetric synthesis of piperidine-2-carboxylic acid and diastereoselective cyclization.

Marinković and Hoffmann reported the radical addition of tertiary amines to α,β-unsaturated lactones using semiconductor photocatalyst powders of TiO_2_ and ZnS (Marinković and Hoffmann, 2001, 2003). They further developed the diastereoselective radical tandem addition-cyclization reaction of (5*R*)-menthyloxyfuran-2(5*H*)-one **9** with aromatic tertiary amines using TiO_2_ or ZnS (Figure 2A) (Marinković and Hoffmann, 2004). Two stereoisomeric tetrahydroquinoline derivatives **10** and **11** were obtained with reasonable diastereoselectivities. The key radical was initially generated *via* the single electron oxidation of *N, N*-dimethylaniline. The stereoselective addition of the alkyl radical to lactone **9** followed by intramolecular radical trapping on the aromatic ring led to the cyclized adduct **10** as the major product.

Ohtani et al. (1990, 2001) reported the deaminocyclization of chiral L-lysine derivatives **12a-c** and 2,6-diaminopimelic acids (DAP) **14a** and **14b** to piperidine derivatives **13** and **15**, respectively (Figure 2B). In the presence of PtO_2_/TiO_2_, the deaminocyclization of L-lysine **12a** gave the piperidine-2-carboxylic acid **13** in moderate enantioselectivity, probably due to the competitive oxidation of the two amino groups of **12a**. Excellent enantioselectivities were achieved by protection of the α-amino group. When the protected L-lysine derivatives **12b** and **12c** were employed, piperidine-2-carboxylic acid **13** was synthesized in 92 and 96% ee, respectively. Additionally, by deaminocyclization of a 1:1 mixture of *racemic* DAP **14a** and *meso* DAP **14b**, preferential production of *trans*-PDC **15** to *cis*-PDC **15** was achieved by changing the catalyst from CdS to PtO_2_/CdS (Figure 2B). The diastereoselectivity was determined at the final photocatalytic hydrogenation of the cyclic imine intermediate formed by oxidative deamination and cyclization. *cis*-**15** would be obtained when the hydrogenation proceeds *via syn*-addition of hydrogen atoms to the C = N bond of the cyclic imine. In contrast, *trans*-**15** is selectively produced when reduction takes place *via* stepwise electron transfer and subsequent stereoselective protonation as illustrated in Figure 2B. Consequently, the opposite diastereoselective preparation of *trans*-PDC and *cis*-PDC was achieved by simply changing the catalyst.

## Photobiocatalysis for Asymmetric Synthesis

Photobiocatalysis employing isolated enzymes or lysates involves three types of electron relay systems (Gulder and Seel, 2019): (1) photosensitizers (metal complexes or semiconductors, etc.), (2) reaction sites (enzymes), and (3) electron mediators such as methylviologen, nicotinamide adenine dinucleotide (phosphate) (NAD(P)^+^/NAD(P)H), and flavin mononucleotide (FMN/FMNH_2_). Excited electrons generated at the photosensitizer are relayed to the reaction site *via* the electron mediators. In addition, sacrificial electron donors (e.g., tertiary amines or water) are required to prevent oxidative self-degradation of the photosensitizers. Successful examples of highly enantioselective reactions using semiconductor photocatalysts have been reported. The asymmetric reduction of alkenes using old yellow enzymes in cooperation with CdSe quantum dots (Burai et al., 2012), gold nanoparticle- loaded TiO_2_ (Au/TiO_2_), or vanadium doped TiO_2_ (Mifsud et al., 2014) has been investigated. Moreover, stereoselective activation of C-H bonds during peroxygenase-catalyzed hydroxylation of alkylbenzenes and alkanes has been achieved using Au/TiO_2_ (Zhang et al., 2017, 2018).

## Conclusion and Outlook

This mini-review focuses on the enantio- and diastereoselective organic reactions occurring in several semiconductor photocatalyses. As mentioned in this review, our group found that enantioselective hydrogenation on TiO_2_ was strongly affected by the surface structure of TiO_2_ (Kohtani et al., 2018). Recently, it was also demonstrated that adsorption of chiral molecules on a specific semiconductor nanoparticle surface (mercury sulfide: HgS) was associated with the growth of chiral semiconductor nanoparticles (Kuno et al., 2018). Thus, one promising strategy may be the use of highly uniform semiconductor nanocrystals with specific exposure of the reactive facets. If these facets could be selectively covered with stable chiral compounds, enantioselective reactions would be greatly enhanced. Therefore, increasing attention should be given to the development of specifically reactive facets on semiconductor materials for stereoselective organic transformations.

## Author Contributions

All authors listed have made a substantial, direct and intellectual contribution to the work, and approved it for publication.

### Conflict of Interest Statement

The authors declare that the research was conducted in the absence of any commercial or financial relationships that could be construed as a potential conflict of interest.
